# The High Prevalence of Anemia in Cambodian Children and Women Cannot Be Satisfactorily Explained by Nutritional Deficiencies or Hemoglobin Disorders

**DOI:** 10.3390/nu8060348

**Published:** 2016-06-07

**Authors:** Frank Tammo Wieringa, Miriam Dahl, Chhoun Chamnan, Etienne Poirot, Khov Kuong, Prak Sophonneary, Muth Sinuon, Valerie Greuffeille, Rathavuth Hong, Jacques Berger, Marjoleine Amma Dijkhuizen, Arnaud Laillou

**Affiliations:** 1 Institute of Research for Development (IRD), UMR Nutripass UM-IRD-SupAgro, Montpellier 3400, France; valerie.greffeuille@ird.fr (V.G.); jacques.berger@ird.fr (J.B.); 2 Department of Fisheries, Post-harvest Technologies and Quality control, Fisheries Administration, MAFF, 186 Preah Norodom Boulevard, Phnom Penh 12000, Cambodia; dahl.miriam@gmail.com (M.D.); chhounchamnan@gmail.com (C.C.); kuong.kh@gmail.com (K.K.); 3 UNICEF, Maternal, Newborn and Child Health and Nutrition section, no11 street 75, Phnom Penh 12202, Cambodia; epoirot@unicef.org (E.P.); alaillou@unicef.org (A.L.); 4 National Nutrition Program, Maternal and Child Health Center, No 31A, Rue de France (St. 47), Phnom Penh 12202, Cambodia; sophonprak@gmail.com; 5 National Center for Parasitology, Entomology and Malaria Control (CNM), Phnom Penh 12202, Cambodia; sinuonm@cnm.gov.kh; 6 ICF International, 530 Gaither Road, Suite 500, Rockville, MD 20850, USA; rathavuth.hong@icfi.com; 7 Nutrition, Exercise and Sports (NEXS), Copenhagen University, Rolighedsvej 25, 1958 Frederiksberg, Copenhagen DK-1958, Denmark; madijkhuizen@gmail.com

**Keywords:** anemia, iron, vitamin A, folic acid, vitamin B12, zinc, hemoglobin disorders, children, women of reproductive age, Cambodia

## Abstract

Background: Anemia is highly prevalent in Cambodian women and children, but data on causes of anemia are scarce. We performed a national micronutrient survey in children and women that was linked to the Cambodian Demographic Health Survey 2014 (CDHS-2014) to assess the prevalence of micronutrient deficiency, hemoglobin disorders and intestinal parasite infection. Methods: One-sixth of households from the CDHS-2014 were selected for a follow-up visit for the micronutrient survey. Households were visited from two weeks to two months after the CDHS-2014 visit. Data on micronutrient status were available for 1512 subjects (792 children and 720 women). Results: Anemia was found in 43% of the women and 53% of the children. Hemoglobin disorders affected >50% of the population, with Hemoglobin-E the most prevalent disorder. Deficiencies of iron (ferritin < 15 g/L), vitamin A (retinol-binding-protein (RBP) < 0.70 mol/L) or vitamin B12 (<150 pmol/L) were not prevalent in the women (<5% for all), whereas 17.8% of the women had low concentrations of folic acid (<10 nmol/L). In the children, the prevalence of iron, vitamin A, vitamin B12 or folic acid deficiency was <10%. Zinc deficiency, hookworm infection and hemoglobinopathy were significantly associated with anemia in children, whereas in the women none of the factors was significantly associated with anemia. Iron deficiency anemia (IDA) was more prevalent in children <2 years, but in older children and women, the prevalence of IDA was <5%. The most prevalent, preventable causes of anemia were hookworm infection and zinc and folic acid deficiency. Over 40% of the anemia was not caused by nutritional factors. Conclusion: The very high prevalence of anemia in Cambodian women and children cannot be explained solely by micronutrient deficiencies and hemoglobin disorders. Micronutrient interventions to improve anemia prevalence are likely to have limited impact in the Cambodian setting. The focus of current interventions to reduce the high prevalence of anemia in children and women should be broadened to include zinc and folic acid as well as effective anti-hookworm measures.

## 1. Introduction

Anemia is characterized by an insufficient concentration of hemoglobin, the protein essential for the transport of oxygen. Hemoglobin is the principal component of red blood cells, erythrocytes, and is synthesized in the bone marrow, with iron being an essential part of the oxygen binding site. A large part of the human population worldwide, especially women of reproductive age and children, is affected by anemia, with an estimated 1.6 billion people being anemic [1].

The World Health Organization has set reference cut-off points for normal populations, and defined that anemia is a public health problem when the prevalence of anemia is >20% in a given population, and a prevalence of >40% indicates a serious health problem [2]. Anemia is a serious public health problem in Cambodia, with the Cambodian Demographic Health Survey 2014 (CDHS-2014) showing that 45% of the women of reproductive age (WRA) and 55% of the children <5 years of age were anemic [3]. Surprisingly, despite significant gains in economic development in Cambodia, the prevalence of anemia in women and children has not changed in the last two decades.

Kassebaum *et al.* [4] showed that, worldwide, iron deficiency anemia (IDA) is the most important contributor to anemia. However, although iron deficiency (ID) is the most important cause of anemia, it is certainly not the only nutrient deficiency causing anemia [2]. Indeed, some controversy exists as to what extent iron deficiency contributes to overall anemia prevalence [1]. Many studies have shown that iron deficiency accounts roughly for only half of the anemia cases [5], but there are wide differences between regions and countries [4]. Unfortunately, anemia is often considered a synonym for iron deficiency, whereas anemia and iron deficiency are distinct, albeit overlapping, conditions. This means that improving iron status in an anemic population will only reduce the prevalence of anemia to a certain, often unknown extent. Other nutrient deficiencies clearly related to the development of anemia are vitamin B12 and folic acid deficiency, although these causes were not included in the analysis by Kasselbaum *et al*. [6]. Dietary intake data from Cambodia showed that recommended daily intakes of iron and folic acid are not met in infants, children and women of reproductive age [7] In addition, diets lacked calcium, zinc, and several B vitamins.

Another important cause of anemia worldwide is hemoglobinopathies. Hemoglobinopathies are genetic disorders affecting hemoglobin synthesis or erythrocyte lifespan. As a result, depending on the type and severity of the disorder, they can cause lower hemoglobin concentrations and hence increase the risk for anemia. There are many different varieties of hemoglobinopathy, with different clinical presentation and specific effects on the hemoglobin protein and/or erythrocyte. Their presence and distribution in a population depends on the genetic background of a population and comprises both homozygotic and the usually milder heterozygotic presentations. In Cambodia, hemoglobin E is the most prevalent hemoglobinopathy, estimated to affect one-third of the population, although national data are lacking [8].

Hence, it appears likely that the current high prevalence of anemia in Cambodia is at least partly caused by iron deficiency. However, two recent studies examining biochemical data for iron status found that iron deficiency is not a major issue in Cambodian women of reproductive age (WRA) [9] nor in school children [10], but that hemoglobinopathies play a much more prominent role in the etiology of anemia. Therefore, there is currently uncertainty on the etiology of the high prevalence of anemia in Cambodia. To answer the question on what causes the high prevalence of anemia in women and children in Cambodia, we collected data on the most common nutrient deficiencies associated with anemia (iron deficiency, vitamin B12 deficiency, folic acid deficiency and vitamin A deficiency), as well as data on hemoglobin disorders, hookworm infection and systemic inflammation during the 2014 Cambodian Micronutrient Survey (CMNS-2014).

## 2. Materials and Methods

### 2.1. Study Design

The 2014 Cambodian Micronutrient Survey (CMNS-2014) followed the 2014 Cambodian Demographic Health Survey (CDHS-2014) but was not an integral part of it; that is, additional data were collected after the data collection of the CDHS-2014 had been done. The CMNS-2014 was implemented in one-sixth of the clusters selected for the CDHS-2014, and data were collected only in women and children <6 years of age. In these clusters, blood, urine, and stool samples were collected from women who had given birth in the five years preceding the survey and from their children aged six to 72 months. Only clusters where data on anemia had been collected by the CDHS-2014 were eligible for inclusion in the CMNS-2014. Hence, from the 611 clusters of the CDHS-2014, 102 clusters were randomly selected for the CMNS-2014, while ensuring that each of the 19 provinces was represented.

### 2.2. Subjects

Subjects were WRA and children from selected households who had participated in the CDHS-2014 in the previous weeks, and who gave informed, written consent to participate in the CMNS-2014. If more than one child from the household fulfilled the selection criteria, all of them were included in the CMNS-2014. Women reporting they were pregnant were excluded, due to the difficulties in interpretation of biochemical values of micronutrient indicators in pregnancy; however, we did not test for pregnancy. Subjects who were ill on the day of the visit were also excluded.

The National Ethical Committee for Health Research (NECHR) from the Ministry of Health, Phnom Penh, Cambodia, reviewed and approved the study protocol (057 NECHR, 11 March 2014). In addition, ethical approval was obtained from the International Review Board of ICF (132989.0.000.KH.DHS.01). The study was carried out following the rules of the Declaration of Helsinki (1975). All household and women were informed verbally and in writing about the aims and procedures of the study, and informed consent was obtained from all women and children, via their mother or guardian approval for these later, before enrollment.

### 2.3. Data Collection

As described above, the CMNS-2014 team visited households between two weeks and two months after the teams of the CDHS-2014. During the visit of the CDHS-2014 team, all households that fell within the inclusion criteria of the micronutrient survey were identified, and contact information of these households was provided to the CMNS-2014 team, including the unique identification number, enabling linking of the CMNS-2014 data to the CDHS-2014 dataset. After checking for the HH code and ID codes, a short questionnaire was taken from the mother/caretaker. Thereafter, anthropometry was measured in the mother and child, and blood (trace element–free, heparine-coated tubes; BioGreiner One, Austria) and urine samples were taken. A container for stool sample collection was also provided with instructions, to be returned the next day. Blood and urine samples were stored on coolpacks (blue ice), and transported in the afternoon to the provincial health center, the stool samples were collected the following day, and also stored in a cool box, and transported to the provincial health center. Blood samples were separated the same afternoon, and plasma and urine samples were frozen at −20 °C at the provincial health center before being transported to Phnom Penh. All samples were transported to Phnom Penh within a week of collection.

### 2.4. Hemoglobin, Hemoglobin Disorders and Biochemical Determination of Micronutrient Status

Hemoglobin concentrations were measured using the Hemocue 3.01 system by the CDHS-2014 teams. Hemoglobin patterns were analyzed using electrophoreses (Minicap, SEBIA) at the Pasteur Institute, Phnom Penh, Cambodia. Criteria for the classification of the hemoglobin patterns the following (Table 1).

Iron status was determined using ferritin (Fer) and soluble transferrin receptor (sTfR) [11]. For vitamin A status, retinol-binding protein (RBP) was used. RBP is the transport protein of retinol in the circulation and is used as a proxy indicator for retinol concentrations [12]. Many indicators of micronutrient status are affected by inflammation, including ferritin and RBP concentrations [13]. Therefore, to be able to correct for the effects of inflammation on these indicators, C-reactive protein (CRP) and α1-acidglycoprotein (AGP) were also measured in all individuals. Inflammation was defined as CRP > 5 mg/L and/or AGP > 1 g/L. Inflammation status was then categorized in four groups based on CRP and AGP levels: no inflammation (normal CRP and AGP), incubation (high CRP and normal AGP), early convalescence (high CRP and AGP), and late convalescence (normal CRP and high AGP). Correction factors were then applied to adjust values of Fer and RBP as described previously [14,15]. Correction factors for ferritin (ferritin-corrected = Fer/correction factor) were 1.30, 1.90 and 1.36 for incubation, early convalescence and late convalescence, respectively. Correction factors for RBP were 0.87, 0.76 and 0.89 for incubation, early convalescence and late convalescence, respectively. Cut-offs used for micronutrient status were as follows: no iron stores was defined as Fer-corrected < 15 µg/L for both women of reproductive age and children, and marginal iron stores as Fer-corrected < 50 µg/L; iron-deficient erythropoiesis (tissue iron deficiency) was defined as sTfR > 8.3 mg/L. Total body iron was calculated using corrected ferritin concentrations and sTfR using the formula of Cook [16]. Plasma zinc concentrations were measured at the National Institute of Nutrition with atomic absorption spectrophotometry, using trace element–free procedures. Vitamin B12 and folic acid were measured at the Pasteur Institute, Phnom Penh, Cambodia, by the Cobas platform, following the manufacturer’s instructions.

### 2.5. Sample Size Considerations

To obtain a precision of 3% at the national level for the prevalence of anemia, a total sample size of 1000 children and 1000 mothers was calculated. This sample size would also give a 5% precision for differences between urban and rural areas. The study was not designed for provincial-level comparisons.

### 2.6. Statistical Analysis

Data entry was done in duplicate using pre-programmed data entry forms in EpiData software (EpiData association, Odense, Denmark). Data was checked for normality using the Kolmogorov-Smirnoff test. Comparisons between regions and urban/rural were done by analysis of covariance (ANCOVA), controlling for age for the children. Prevalence of deficiency was analyzed using binary logistic analysis, correcting for sampling procedures. Differences in plasma concentrations were tested using analysis of co-variance. *p*-values < 0.05 were considered statistically significant. Data was analyzed using SPSS software (Version 20.0, IBM, New York, NY, USA).

## 3. Results

Data on micronutrient status (collected by the CMNS-2014) were available for 1526 subjects (801 children and 725 women, Table 2). Data on hemoglobin concentrations (collected by the CDHS-2014) were available for 1102 subjects (632 children and 470 women, Table 2), but as the two surveys did not always include the same subjects, combined data on hemoglobin concentrations and micronutrient status were available for 856 subjects only (415 children and 441 women, Table 3). Numbers differ slightly between the different indicators of micronutrient status as indicators were measured in different laboratories, and sometimes not enough sample was available to complete all analyses (Table 2).

The prevalence of anemia in the subgroup of children and the mothers participating in the CMNS-2014 was high (53.9% and 43.6%, respectively), but the prevalence was not different from the anemia prevalence in the overall study population of the CDHS 2014 (55.5% and 45.4%, respectively). Evidence of sub-clinical inflammation (one or more increased acute phase protein concentrations) was present in 39.8% of the children and 41.1% of the women, with most subjects being in the late convalescence phase (30.2% and 29.8% of the children and women, respectively, Table 2).

The prevalence of anemia differed significantly over the type of hemoglobin pattern (Table 3). Hemoglobin E patterns were the most common type of hemoglobinopathy, with heterozygous HbE giving overall (children and women combined) a 1.5× higher risk for anemia, whereas homozygous HbE gave a 7.0× higher risk for anemia (Table 3) as compared to a normal hemoglobin pattern. However, the effect of being a carrier of HbE (*i.e.*, heterozygote) on the risk for anemia was stronger in children than in women (Table 4), with heterozygote HbE in women not being significantly associated with anemia.

We further explored associations between several nutritional deficiencies and anemia. In children, anemia was only associated with zinc deficiency, albeit it was much stronger in children with a normal hemoglobin pattern than in children with an abnormal hemoglobin pattern (Table 4). Although odds ratios (ORs) for almost all other biomarkers of micronutrient status were >1, none of them reached statistical significance. For most indicators of micronutrient status, there was a significant interaction (*p* < 0.10) with hemoglobinopathy, indicating that biomarkers for micronutrient status have a different association with anemia in children with or without hemoglobinopathy.

In the women, none of the biomarkers for micronutrient status was significantly associated with anemia, although there were tendencies for vitamin A, zinc and folic acid deficiency (*p* < 0.2). As for the children, associations between biomarkers of micronutrient status were often markedly different between subjects with a normal hemoglobin pattern and subjects with evidence of hemoglobinopathy (Table 5).

To further explore the contribution of iron deficiency to the high anemia prevalence in children, we divided the children into different age groups (Table 6). Iron deficiency anemia, defined as anemia combined with a ferritin concentration <15 µg/L, was more prevalent in children <3 years of age, but the prevalence declined rapidly after two years of age. At the same time, the prevalence of children with no anemia also increased significantly, indeed, more than doubling from children <2 years of age to children >3 years of age.

When assessing the strength of predictors of anemia in a full model (Table 7), hemoglobinopathy, zinc deficiency and hookworm infection were all significantly associated with anemia in the children. Surprisingly, in the women, none of the factors was significantly associated with anemia, although there was a slight tendency for hemoglobinopathy and folic acid deficiency to be related to anemia (*p* < 0.2).

**Figure 1:**
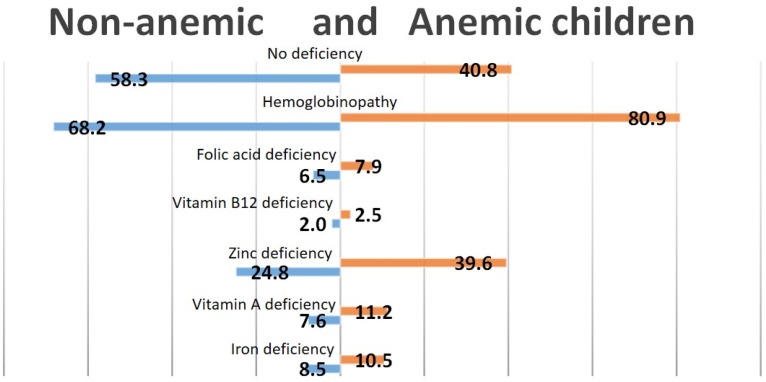
Prevalence of nutritional deficiencies and hemoglobinopathy in anemic and non-anemic children.

**Figure 2:**
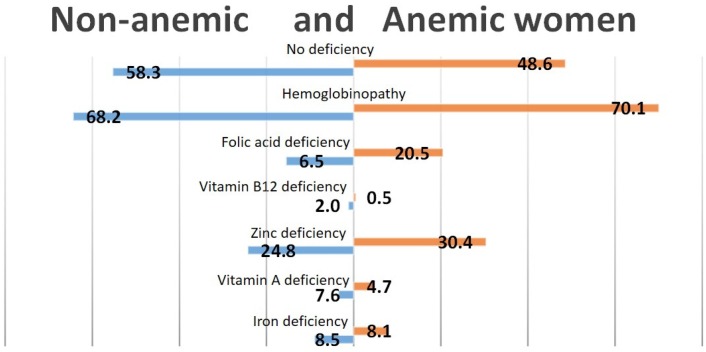
Prevalence of nutritional deficiencies and hemoglobinopathy in anemic and non-anemic women.

Even though almost none of the nutritional factors were significantly associated with anemia, we tried to assess the contribution of each nutritional factor and hemoglobinopathies to the high prevalence of anemia in Cambodian children (Figure 1) and women (Figure 2). As shown in Figure 1, over 40% of the children with anemia were not deficient in iron, zinc, vitamin B12, folic acid or vitamin A. The largest contributor to nutritional anemia in children was zinc deficiency, with almost 40% of the children having both anemia and zinc deficiency, compared to 24.8% of the non-anemic children. Only 10.5% of the children with anemia could be classified as having IDA.

Almost 50% of the anemic women were not deficient in iron, vitamin A, folic acid, vitamin B12 or zinc (Figure 2). Only 8.1% of the anemic women were iron-deficient. As for the children, zinc deficiency was the largest contributor to nutritional anemia, with almost one in three of the women with anemia also having severe zinc deficiency. In addition, there was a high prevalence of folic acid deficiency, with one in five of the anemic women being deficient in folic acid.

## 4. Discussion

Anemia is highly prevalent in Cambodia, and a serious health problem according to WHO guidelines, with >40% of the women and >50% of the children affected. Iron deficiency was assumed to be an important cause. However, in the present paper, we show that frank iron deficiency, *i.e.*, ferritin concentrations <15 µg/L, is not very prevalent in Cambodian children or women, and hence not an important contributor to the overall anemia prevalence. An exception is children <2 years of age. In this age group, iron deficiency anemia (anemia and a ferritin concentration <15 µg/L) is prevalent, with ~10% of the children affected. Moreover, deficiencies of folic acid, vitamin B12 or vitamin A were not significantly associated with anemia in the children or the women whereas, surprisingly, zinc deficiency was related to anemia, even though only in the children.

The prevalence of frank iron deficiency (ferritin <15 µg/L) or marginal iron status (ferritin <50 µg/L) is not very different from a recent survey from Vietnam, which reported iron deficiency in 12.9% of the women and IDA in only 3.2% of the women [17]. However, in contrast to the study in Vietnam, the prevalence of anemia was much higher in Cambodia (43.6%) than in Vietnam (9.1%) [17]. Another biomarker for iron status, sTfR, was also not associated with anemia, even though the prevalence of tissue iron deficiency (sTfR > 8.3 mg/L) was much higher than frank iron deficiency. Even in Cambodian women with a normal hemoglobin pattern, anemia prevalence (38.4%) was much higher than in Vietnam, suggesting that differences in hemoglobinopathy prevalence between Cambodia and Vietnam cannot explain this striking difference in anemia prevalence. Indeed, as reported before, hemoglobinopathies are highly prevalent in Cambodia [8], affecting >50% of the population, and are a much more important contributor to the high prevalence of anemia than iron deficiency [18]. However, whereas carriers of the HbE mutation, alone or in combination with β-thallasemia, had a higher prevalence of anemia than subjects with a normal Hb pattern (54.3% *vs*. 38.4%), many other forms of hemoglobinopathies had no significantly increased prevalence of anemia. Hemoglobinopathies did, however, interact significantly with most biomarkers for micronutrient status, suggesting that many cut-offs for these biomarkers might not be valid in subjects with hemoglobinopathy.

The risk factors found in this study for anemia in Cambodian children included causes often associated with anemia: hemoglobinopathy and hookworm infection. In the women, however, none of the nutritional factors was significantly associated with anemia. This raises fundamental questions on what causes the high prevalence of anemia in Cambodian women.

The significant association of zinc deficiency with anemia in children in the present study is intriguing. Zinc is usually not one of the nutrients associated with anemia, although there are reports that zinc deficiency might increase oxidative stress and thereby reduce the lifespan of the erythrocyte, causing anemia [19]. Another mechanism through which zinc deficiency might affect hemoglobin concentrations is lower erytropoeitin concentrations. Regardless of the underlying mechanism, more research is needed to test whether improving the zinc status of the Cambodian population might result in reducing the high prevalence of anemia. Interventions to reduce the high prevalence of zinc deficiency in Cambodia are warranted anyway, given the role of zinc deficiency in infectious diseases, especially diarrheal disease, and linear growth retardation.

Even though folic acid deficiency was not significantly associated with anemia, 20% of the women with anemia had folic acid deficiency. Folic acid deficiency during pregnancy is strongly associated with prematurity and neural tube defects [20]. Therefore, efforts to improve the folic acid status of women of reproductive age in Cambodia are urgently needed.

In our study, we might have overestimated the prevalence of abnormal hemoglobin patterns in children. In children, HbF often persists well into the second year of life. However, setting less stringent limits for HbA did not result in different outcomes of the analysis of factors contributing to anemia. Another limitation in our study is that hemoglobin concentrations were measured by the Hemocue system during the CDHS-2014 survey, whereas micronutrient status was measured during the CMNS-2014, meaning that hemoglobin concentrations were measured several weeks before the measurement of micronutrient status. Although hemoglobin concentrations are not likely to change substantially within a few weeks, we would have preferred to measure iron status and hemoglobin concentrations in the same samples. Unfortunately, this was not possible due to logistical constraints. Also, the set of subjects for whom hemoglobin data were available was not identical to the set of subjects for whom we had data on micronutrient status, thereby reducing the number of subjects available for statistical analysis on the causes of anemia, and hence the power of the study.

However, despite these limitations, we are confident in our finding that neither nutritional factors nor the high prevalence of hemoglobinopathies (especially HbE) can sufficiently explain the high prevalence of anemia in Cambodian children and women of reproductive age. This could mean that the cut-off for anemia is not valid for the Cambodian population. However, research from Indonesia concluded that the current WHO cut-offs for anemia were valid in a South Asian context [21]; hence, it is unlikely that the current cut-offs need to be adjusted for Cambodia. It is tempting to attribute part of the anemia to zinc deficiency, given the strong association between zinc deficiency and anemia in children. However, the exact physiological pathways in which zinc deficiency could lead to anemia are not clear, even though zinc status has been shown to affect erytropoeisis [22]. If the found association between zinc deficiency and anemia is proven not to be directly causal in the Cambodian context, then >70% of the anemia in Cambodian children is due to factors that are not amendable (hemoglobinopathy) or unknown. Another possibility is that rare causes of anemia play a role, such as toxins, kidney disease, other metabolic conditions, as well as environmental pollutants. Also, chronic immune activation, chronic diseases causing anemia, and perhaps rare infectious events that may have been overlooked should be considered, although in the present study, inflammation was measured and taken into account. Clearly more research is needed to elucidate the etiology of anemia in Cambodia, in order to be able to effectively address the alarmingly high prevalence of anemia in this population, as iron supplementation strategies have not been able to effectively address this problem until now, which is understandable, in retrospect, with the results of the present study.

## 5. Conclusions

In Cambodia, anemia is serious public health concern, affecting over 50% of the children under the age of 5 years. However this very high prevalence of anemia in Cambodian women and children cannot be explained solely by micronutrient deficiencies and hemoglobin disorders. Micronutrient interventions to improve anemia prevalence are likely to have limited impact in the Cambodian setting. Iron deficiency prevalence was low in children above 2 years of age and in women. The focus of current interventions to reduce the high prevalence of anemia in children and women should be broadened to include zinc and folic acid for women of reproductive age, as well as effective anti-hookworm measures.

## Figures and Tables

**Table 1:** Classification criteria for hemoglobin patterns obtained by electrophoresis.

Hemoglobin Patterns	Children	Women
Normal hemoglobin	<2 years: HbA > 70%, HbF ≤ 30%, HbA2 < 2%	HbA >95.5%, HbA2 2.0%–3.5%, HbF < 1%
	2–5 years: HbA > 95.5%, HbA2 2.0%–3.5%, HbF <5%	
HbE-heterozygote	HbE 20%–30%	HbE 20%–30%
HbE-homozygote	HbE > 85%, HbF < 15%	HbE > 85%, HbF < 15%
HbE-β-thalassemie	HbF 30%–65%, HbE 40%–60%, HbA < 10%	HbF 30%–65%, HbE 40%–60%, HbA < 10%
β-thalassemie_heteroz.	HbA2 3.5%–8%, HbF 2.0%–7%	HbA2 3.5%–8%, HbF 2.0%–7%
Other	None of above	None of above

**Table 2:** General characteristics and micronutrient status of the study population ^1^.

	Children	Women
*N*	632	470
Age (±SD), years	3.1 (±1.5)	30.0 (±6.5)
Hemoglobin (±SD), g/L	10.8 (±1.3)	11.9 (±1.3)
Anemic (%)	53.9%	43.6%
Hemoglobin pattern (N)	686	709
HbA2 >95.5% (normal)	23.8%	33.2%
Heterozygote HbE	26.7%	28.4%
Homozygote HbE	3.1%	5.9%
HbE-β-thalassemia	0.1%	0.0%
B-Thalassemia heterozygote	1.6%	1.1%
Other	49.2%	31.4%
*N*	792	720
Ferritin (IQR) uncorrected, µg/L ^1^	64.5 (35.8–114.9)	65.1 (37.9–161.1)
Ferritin (IQR) corrected, µg/L	57.2 (31.1–96.4)	58.9 (32.3–97.3)
<15 µg/L	9.1%	6.9%
<50 µg/L	44.1%	42.6%
sTfR (IQR), mg/L ^1^	5.62–15.09)	6.3 (4.34–11.42)
>8.3 mg/L	51.2%	37.2%
Total body iron (IQR), mg/kg	5.65 (2.92–8.18)	6.79 (4.09–8.86)
<0 mg/kg	8.3%	4.3%
<4 mg/kg	24.7%	20.0%
RBP (IQR), µmol/L ^1^	1.40 (1.02–2.40)	2.20 (1.49–3.57)
<0.70 µmol/L	8.9%	3.7%
CRP (IQR), mg/L ^1^	0.19–1.44)	0.69 (0.31–1.89)
>5 mg/l	11.1%	9.7%
AGP (IQR), g/L ^1^	0.78 (0.48–1.55)	0.69 (0.47–1.42)
>1 mg/L	38.8%	35.8%
*N*	780	725
Vitamin B12 (IQR), pmol/L	415 (306–592)	491 (356–683)
<150 pmol/L	1.9%	1.0%
Folic acid (IQR), nmol/L	20.2 (14.3–29.0)	14.1 (10.7–18.8)
<10 nmol/L	7.9%	17.8%
*N*	656	720
Zinc (± SD), µmol/L	8.9 (±2.5)	9.2 (±2.4)
<9.9 µmol/L	67.5%	62.8%
<7.65 µmol/L	33.7%	26.3%
*N*	474	331
Hookworm infection	8.5%	17.0%

^1^ Values are as mean (±SD) when the distribution was normal, otherwise as medians (IQR). Abbreviations: sTfR = soluble transferrin receptor, RBP = retinol-binding protein; CRP = C-reactive protein, AGP = α1-acidglycoprotein. Ferritin and RBP were corrected for inflammation as described in the Methods section.

**Table 3:** Anemia prevalence and hemoglobinopathy in children and women of reproductive age ^1,2^.

	Children (*n* = 415)			Women (*n* = 441)		
	Anemia	Contribution to Overall Anemia	OR Anemia (95% CI)^3^	Anemia	Contribution to Overall Anemia	OR Anemia (95% CI)^3^
Normal Hb pattern (*n*)	37.4% (*n* = 40) ^a^	19.1%	Ref	38.4% (*n* = 56) ^a^	29.9%	Ref
HbE heterozygote (*n*)	54.3% (*n* = 57) ^a,b^	27.3%	**1.99 (1.15**–**3.44)**	41.7% (*n* = 53) ^a^	28.3%	1.19 (0.73–1.93)
HbE homozygote (*n*)	93.8% (*n* = 15) ^b^	7.2%	**25.13 (3.20**–**197.5)**	73.1 (*n* = 19) ^b^	10.2%	**4.31 (1.70**–**10.9)**
HbE-β thalassemia (*n*)	100% (*n* = 1) ^a,b^	0.5%	-	-	0%	-
β thalassemie heterozygote (*n*)	100% (*n* = 4) ^a,b^	1.9%	-	100% (*n* = 5) ^a,b^	2.7%	-
Other forms Hb.pathy (*n*)	49.2% (*n* = 92) ^a^	44%	1.62 (1.00–2.64)	39.4% (*n* = 54) ^a^	28.9%	1.03 (0.64–1.67)

^1^ Hemoglobin patterns were classified according to the definitions given in Table 1; ^2^ Columns with different letters are significantly different from each other; ^3^ Odd ratios (OR) significantly different from reference (normal hemoglobin pattern) are given in bold.

**Table 4:** Unadjusted univariate odds ratios of iron and other vitamin and mineral deficiencies to anemia in children (*N* = 400) ^1^.

	Normal Hb Pattern	Abnormal Hb Pattern	Overall (*N* = 405)	P_interaction_ Hbpathy × Nutrient
No iron stores (Fer < 15 µg/L)	1.26 (0.37–4.27)	1.28 (0.55–2.98)	1.22 (0.67–2.25)	0.39
Marginal iron status (Fer < 50 µg/L)	1.63 (0.72–3.67)	1.17 (0.73–1.85)	1.16 (0.81–1.65)	**0.08**
Tissue iron deficiency (sTfR > 8.3 mg/L)	1.61 (0.72–3.59)	1.12 (0.71–1.77)	1.30 (0.88–1.92)	**0.06**
Vitamin A deficiency (RBP < 0.70 µmol/L)	1.88 (0.56–6.29)	1.86 (0.73–4.75)	1.70 (0.82–3.51)	**0.10**
Marginal vit. A status (RBP < 1.05 µmol/L)	1.17 (0.48–2.87)	1.26 (0.73–2.15)	1.20 (0.76–1.89)	0.12
Zinc deficiency (<7.65 µmol/L)	**3.25 (1.26**–**8.34)**	**1.76 (1.05**–**2.95)**	**2.07 (1.32**–**3.26)**	**0.004**
Vitamin B12 (<150 pmol/L)	-	2.30 (0.44–12.1)	1.07 (0.30–3.75)	0.23
Folic Acid (<10 mmol/L)	3.18 (0.72–14.2)	0.90 (0.40–2.00)	1.22 (0.60–2.47)	0.87

^1^ Odd ratios (OR) significantly different from reference (normal hemoglobin pattern) and significant interactions (*p* < 0.1) are given in bold.

**Table 5:** Unadjusted univariate odds ratios of iron and other vitamin and mineral deficiencies to anemia in women (*N* = 435).

	Normal Hb Pattern	Abnormal Hb Pattern	Overall (*N* = 435)	P_interaction_ Hbpathy × Nutrient
No iron stores (Fer <15 µg/L)	0.67 (0.17–2.70)	0.91 (0.40–2.06)	0.89 (0.45–1.75)	0.80
Marginal iron status (Fer <50 µg/L)	1.01 (0.51–1.98)	0.85 (0.53–1.37)	0.89 (0.60–1.30)	0.69
Tissue iron deficiency (sTfR >8.3 mg/L)	1.80 (0.87–3.72)	0.91 (0.57–1.45)	1.19 (0.81–1.75)	0.64
Vitamin A deficiency (RBP <0.70 µmol/L)	0.62 (0.12–3.28)	2.23 (0.64–7.80)	1.37 (0.54–3.53)	0.31
Marginal vit. A status (RBP <1.05 µmol/L)	0.86 (0.27–2.71)	1.97 (0.85–4.54)	1.48 (0.77–2.85)	0.14
Zinc deficiency (<7.65 µmol/L)	1.58 (0.73–3.43)	1.16 (0.69–1.94)	1.36 (0.89–2.07)	0.25
Vitamin B12 (<150 pmol/L)	-	1.24 (0.08–20.1)	0.45 (0.05–4.36)	0.72
Folic Acid (<10 nmol/L)	1.01 (0.41–2.52)	1.57 (0.86–2.84)	1.41 (0.87–2.29)	0.12

**Table 6:** Prevalence of anemia, iron deficiency and iron deficiency anemia by age group in Cambodian children.

		Age (Months)			
	6–11	12–23	24–36	36–48	>48
*N*	21	76	99	102	192
No anemia	28.6% ^a,b^	26.3% ^b^	47.5% ^a,c^	57.8% ^c^	59.4% ^c^
IDA ^1^	9.5%	7.9%	7.1%	3.9%	3.1%
Anemia, non-ID	61.9% ^a,b^	65.8% ^b^	45.5% ^a,c^	38.2% ^c^	37.5% ^c^

^1^ IDA = iron deficiency anemia, defined as anemia combined with a serum ferritin concentration <15 µg/L. ID = iron deficiency defined as a serum ferritin concentration < 15 g/L; ^2^ Rows with different letters are significantly different from each others. Chi-square analysis with correction for multiple comparisons, *p* < 0.05.

**Table 7:** Relative risks for anemia in children (*N* = 305) and women (*N* = 363) in a full model^1^.

	Children ^1^		Women ^2^	
	B (95% CI)	*p*	B (95% CI)	*p*
Hemoglobinopathy (yes/no)	**1.90 (1.07**–**3.37)**	**0.03**	1.36 (0.87–2.14)	0.18
Iron deficiency (ferritin < 15 µg/L)	1.72 (0.74–3.98)	0.20	0.82 (0.35–1.87)	0.63
Tissue Fe deficiency (sTfR > 8.3 mg/L)	1.05 (0.60–1.82)	0.87	1.12 (0.72–1.75)	0.62
Vitamin A deficiency (<0.70 µmol/L)	1.50 (0.58–3.85)	0.40	1.34 (0.48–3.78)	0.58
Zinc deficiency (<9.95)	**1.85 (1.10**–**3.13)**	**0.02**	1.15 (0.72–1.83)	0.56
Hookworm infection	**3.09 (1.28**–**7.47)**	**0.01**	1.08 (0.62–1.87)	0.79
Vitamin B12 deficiency (<150 pmol/L)	1.25 (0.22–7.12)	0.80	0.44 (0.05–4.39)	0.49
Folic acid deficiency (<10 nmol/L)	1.59 (0.69–3.67)	0.28	1.54 (0.88–2.69)	0.13

^1^ Risk factors significantly associated with anemia are given in bold; ^2^ Binary logistic regression, Nagelkerke *R*^2^ = 0.095; ^3^ Binary logistic regression, Nagelkerke *R*^2^ = 0.038.
